# Social media intervention for promoting breastfeeding among WIC participants

**DOI:** 10.1002/fsn3.3620

**Published:** 2023-08-23

**Authors:** Louisiana M. Sanchez, Sung‐Yeon Park, Taya Kohnen, Bret Sarnquist, Hyo Jin (Jean) Jeon, Michelle Granner, Kelly Morning, Patricia MacNeil, Olivia Deavers, Valery Soto, Elizabeth Christiansen

**Affiliations:** ^1^ School of Public Health University of Nevada Reno Nevada USA; ^2^ WIC Breastfeeding Support Campaign, School of Public Health University of Nevada Reno Nevada USA; ^3^ Division of Public and Behavioral Health Nevada Department of Health and Human Services Carson City Nevada USA; ^4^ Department of Marketing College of Business, University of Nevada Reno Nevada USA; ^5^ Nevada Public Health Training Center University of Nevada Reno Nevada USA; ^6^ Supplemental Nutrition and Safety Programs, Food & Nutrition Service, USDA Virginia Alexandria USA; ^7^ Center for Program Evaluation, School of Public Health University of Nevada Nevada Reno USA

**Keywords:** attitude, breast feeding, Facebook, self‐efficacy, social media, social support, WIC

## Abstract

Social media have emerged as a promising communication channel for promoting breastfeeding among a new generation of mothers. Yet, there is no published study reporting the effects of a large‐scale social media intervention on key breastfeeding‐related perceptions, attitudes, and behaviors. As a component of its breastfeeding promotion campaign, the Women, Infants, and Children (WIC) program implemented a 12‐month intervention using Facebook and Instagram and subsequently evaluated the outcomes by surveying WIC‐participating women (*N* = 832) twice, immediately before and after the intervention. Based on their level of exposure to the intervention messages, the women were retrospectively classified into two groups, resulting in a two‐group (no–low exposure vs. medium–high exposure) quasi‐experiment. Women in the medium–high exposure group, in comparison with women in the no–low exposure group, exhibited higher campaign awareness (*p* < .001), visits to the campaign website (*p* < .001), and engagement with the website content (*p* < .001). They also reported more positive breastfeeding attitudes (*M* = 17.26 vs. *M* = 16.51, *p* < .05), self‐efficacy (*M* = 54.48 vs. *M* = 49.94, *p* < .01), and social support (*M* = 27.37 vs. *M* = 25.11, *p* < .001). But they did not differ from women in the no–low exposure group in breastfeeding initiation (*p* > .05) and duration (*p* > .05). In conclusion, a social media‐based intervention resulted in more positive breastfeeding attitudes, higher self‐efficacy, and higher perceived social support. Future studies need to investigate the optimal level of intervention message dosage that prompts significant behavioral changes.

## Graphical abstract

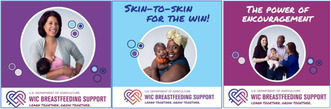

The Special Supplemental Nutrition Program for Women, Infants, and Children (WIC) is one of the largest federal nutrition programs, and it serves more than 42% of all infants in the U.S. (Food and Nutrition Services, 2022). In 1997, the USDA Food and Nutrition Service (FNS) launched the “Loving Support Makes Breastfeeding Work” campaign. Adopting social marketing principles, the campaign emphasized the support of family, friends, health care providers, employers, and the community while directly helping WIC‐participating women with their breastfeeding needs. After 20 years, the campaign was credited with rising breastfeeding initiation and duration rates among WIC participants and changing the social, cultural, and regulatory environments of breastfeeding mothers (Perez‐Escamilla, 2012).

In 2018, USDA FNS introduced the second generation of the campaign under a new slogan, “WIC Breastfeeding Support. Learn Together, Grow Together.” One central component of the renewed campaign was a social media‐based program where State WIC agencies disseminated breastfeeding promotion posts–some informational and others motivational–on popular social media platforms. The focus on social media was born from expert recommendations based on a comprehensive evaluation of the “Loving Support Makes Breastfeeding Work” campaign (The National Academies of Science, Engineering, and Medicine, 2011). After issuing a request for applications open to all 89 WIC agencies operating in U.S. and territories, FNS selected six State WIC Agencies (SAs) and one WIC Indian Tribal Organization (ITO) based on their applications' enthusiasm for the campaign, feasibility, staff qualifications, and geographic considerations. The social media intervention was implemented for one year between August 1, 2020 and July 31, 2021.

The intervention's goal was *to provide breastfeeding promotion and support and build a supportive breastfeeding environment as part of the nutrition education offered to WIC program participants*. Under this goal, several objectives related to the social media intervention were established, some pertaining to communication‐related outcomes such as WIC participants' campaign awareness and use of the WIC breastfeeding support website and others about breastfeeding‐related attitudes and behaviors. This study reports the results of the social media intervention by analyzing the pretest and posttest survey data collected from WIC participants in the seven SAs/ITO.

## 1 PROMOTING BREASTFEEDING VIA SOCIAL MEDIA

With more than 70% of U.S. adults using at least one social media site, social media has become a primary channel for Americans to connect with others and find information (Auxier et al., 2019). In this broader social context, social media have also become an important tool in health promotion (Freeman et al., 2015; Shi et al., 2018). Social media enable greater information sharing and opportunities for community building through an internet‐mediated dialogue that allows users to create content through posts and online discussions (Stellefson et al., 2020). Commonly used social media platforms for nutrition interventions include Facebook, blogs, and forums (Nour et al., 2017).

Breastfeeding advocates have long been urging the use of social media as a communication channel with the new generation of mothers who grew up with the technology (Wolynn, 2012). So far, only a few studies have examined social media‐based breastfeeding promotion programs (e.g., Bahkali et al., 2015; Ferrell et al., 2017). For instance, Breastfeed4Ghana was a social media‐based campaign that successfully demonstrated the feasibility and acceptability of the campaign among never‐married and college‐educated young adults in Ghana (Harding et al., 2020).

Despite the initial success, social media‐based health interventions have many unique challenges, including how to determine the target audiences' exposure to the intervention messages and the dosage. Researchers noted that, often, there were mismatches between those the communication campaign targeted and those it reached (Niederdeppe, 2014). For example, Breastfeed4Ghana social media campaign attracted a population of young and highly educated nonparents, not representative of Ghanaian adults of child‐bearing age (Harding et al., 2020). Moreover, the task of measuring message exposure is less than straightforward (Hornik, 2016). Another breastfeeding social media campaign in Saudi Arabia reported a high number of campaign message impressions among Twitter users but did not conduct a rigorous outcome evaluation among the target audience (Bahkali et al., 2015). Additionally, the speed and dynamism of media message diffusion through social media could produce recall bias among participants, and using social media to deliver specific campaign messages requires thoughtful planning and careful execution (Niederdeppe, 2016).

### 1.1 Attitude, self‐efficacy, and social support in breastfeeding promotion

Research to date has identified attitude and self‐efficacy as two key psychological constructs in breastfeeding promotion. For example, adolescent mothers with more positive breastfeeding attitudes were more likely to initiate breastfeeding (Mossman et al., 2008). Similarly, more positive breastfeeding attitudes among WIC‐participating mothers were related to higher breastfeeding initiation rates, longer duration, and later formula initiation (McCann et al., 2007).

Moreover, an association exists between high breastfeeding self‐efficacy, longer duration of breastfeeding, and higher levels of exclusive breastfeeding (Schlickau & Wilson, 2005). Higher levels of breastfeeding self‐efficacy predicted longer breastfeeding duration among mothers of African descent (McCarter‐Spaulding & Gore, 2009). Similarly, low‐income mothers' high breastfeeding self‐efficacy scores were associated with exclusive breastfeeding (Glassman et al., 2014).

In addition, social support can be particularly helpful for low‐income women. Because low‐income women are also likely to have lower social support and fewer resources (Grassley, 2010; Hudson et al., 2016), breastfeeding support for WIC participants can be crucial in improving breastfeeding‐related attitudes and behaviors among the population. In one study, receiving social support from women's social networks through participation and encouragement was positively associated with breastfeeding self‐efficacy (Maleki‐Saghooni et al., 2020). In another study, social support was also positively associated with breastfeeding self‐efficacy and attitude toward breastfeeding (Mercan & Tari Selcuk, 2021).

**FIGURE 1:**
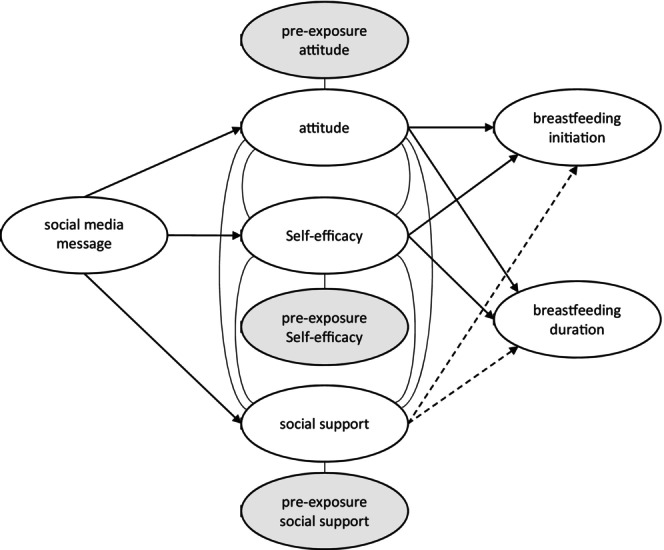
The effects of exposure to social media messages on breastfeeding attitude, self‐efficacy, social support, breastfeeding initiation, and retention.

In sum, breastfeeding attitude, self‐efficacy, and social support are important outcomes to measure when looking at the influence of breastfeeding promotion programs on breastfeeding behaviors. Figure 1 illustrates a conceptual model exploring the effects of social media breastfeeding support messages on the three perceptual/attitudinal variables (i.e., breastfeeding attitude, self‐efficacy, and social support) and two behavioral outcomes (i.e., breastfeeding initiation and duration). The model identifies pre‐exposure breastfeeding attitude, self‐efficacy, and social support as potential confounding variables for their respective postexposure measure.

### 1.2 Research questions and hypotheses

Because the social media intervention was part of the first full‐scale effort by FNS to launch the “Learn Together, Grow Together” campaign, the objectives included higher awareness of the campaign and increased use of the WIC Breastfeeding Support website. These hypotheses (H) directly addressed the campaign‐specific objectives:

Exposure to WIC Breastfeeding Support social media intervention will raise awareness of the WIC Breastfeeding Support Campaign.

Exposure to the WIC Breastfeeding Support social media intervention will increase visits to the WIC Breastfeeding Support website.

Exposure to WIC Breastfeeding Support social media intervention will increase engagement with the content on the WIC Breastfeeding Support website.

In addition, the social media intervention had objectives regarding breastfeeding‐related perceptions and attitudes. These hypotheses directly addressed the objectives:

Exposure to the WIC Breastfeeding Support social media intervention will generate a more positive attitude toward breastfeeding.

Exposure to the WIC Breastfeeding Support social media intervention will generate higher breastfeeding self‐efficacy.

Exposure to the WIC Breastfeeding Support social media intervention will generate higher perceived social support.

Lastly, the effects of social media intervention on breastfeeding behaviors were examined. The National Breastfeeding Awareness Campaign (NBAC), conducted by the U.S. Department of Health and Human Services Office on Women's Health between 2004 and 2006, was a multimedia national campaign that deployed television, radio, newspapers, magazines, mass transit shelters, and a website. Although the NBAC demonstrated the potential of using online media as part of a larger effort by garnering a large number of visitors to the website, it did not perform outreach through social media channels. Hence, two research questions rather than hypotheses were generated here to examine the effects of social media intervention on breastfeeding behaviors. Additionally, we explored the indirect effects of social media intervention on breastfeeding initiation and duration via breastfeeding‐related perceptions and attitudes.

Will exposure to the WIC Breastfeeding Support social media intervention generate a higher breastfeeding initiation rate?.

Will exposure to the WIC Breastfeeding Support social media intervention generate a longer breastfeeding duration?

Will exposure to the WIC Breastfeeding Support social media intervention generate a higher breastfeeding initiation rate via a more positive breastfeeding attitude, higher self‐efficacy, or higher perceived social support?

Will exposure to the WIC Breastfeeding Support social media intervention generate a longer breastfeeding duration via a more positive breastfeeding attitude, higher self‐efficacy, or higher perceived social support?.

## 2 METHODS

### 2.1 Intervention

The social media intervention involved the two most popular social media platforms, Facebook and Instagram. The SA/ITOs either used their existing Facebook and Instagram pages or set up new pages for the intervention. Leading up to the launch of the intervention in August 2020, SA/ITOs promoted the social media pages through their regular communication channels with clients (e.g., text messages, flyers, posters, etc.) Five standard social media posts per week, a total of 260 different posts, were distributed via the two platforms over the 12‐month intervention period, and the same posts were made on Facebook and Instagram on the same schedule. Many of the posts explicitly encouraged their viewers to interact with the posts by liking, commenting, and sharing with others. SA/ITOs also had designated personnel who responded to inquiries and moderated conversations when warranted.

### 2.2 Design

A two‐group pretest–posttest design was employed to examine the effects of the social media intervention. Baseline pretest surveys were collected immediately before the beginning of the intervention program. Those who participated in the pretest surveys were contacted again by the end of the program to complete the posttest survey. The two groups (high vs. low exposure) were constructed retrospectively based on their level of exposure to social media messages.

### 2.3 Procedure

Baseline pretest surveys were administered to prenatal or breastfeeding, active WIC participants by SA/ITO site staff between June 15 and July 31, 2020. WIC staff used a combination of text messages and mailouts/postcards to share the online pretest survey link. Contact information of pretest participants was retained by SA/ITO site staff, who used the list to contact them again for posttest surveys between June 14 and July 31, 2021. This study was deemed exempt by the University of Nevada, Reno Institutional Review Board under federal regulation 45 46.101 (b) CFR. Reference: www.hhs.gov/ohrp/humansubjects/guidance/45cfr46.html

### 2.4 Participants

The pretest was taken by 2705 individuals, but only about one‐third of them (*n* = 832, 31%) completed the posttest, resulting in the matched pretest–posttest dataset containing 832 cases. The respondents' ages ranged from 17 to 44 years, with a mean age of 29.7 years. The largest percentage of respondents were 30–34 years old (30%) and were high school graduates or had a GED (44%). Homemakers/stay‐at‐home moms made up 41% of the sample, 27% were employed full‐time, and 19% were unemployed. More participants identified themselves as an ethnic or racial minority (61%) than as a non‐Hispanic Caucasian (39%). Forty‐three percent were married, 31% were single or never married, and 20% lived together with a partner.

### 2.5 Measures

#### 2.5.1 Intervention message exposure

The posttest survey contained three questions to assess participants' exposure to intervention messages. The first question asked whether they had seen WIC Breastfeeding Support messages during the past 12 months. Only those who answered “yes” to the first question were presented with the second question asking where they had seen the messages. Only those who checked “Facebook” and/or “Instagram” among 15 different sources were asked the third question about how often they had seen the WIC Breastfeeding Support messages. Those who answered “sometimes” and “often” to the third question were classified into the “medium‐high exposure” group. All others who participated in the pretest and posttest were classified into the “no‐low exposure” group.

#### 2.5.2 Campaign awareness

Adopting the method used in a large‐scale health promotion campaign (Potter et al., 2008), we used two indicators, the percentages of participants who accurately identified the campaign tagline and logo, respectively, to measure campaign awareness. Each question presented four choices, one of which was the correct answer.

#### 2.5.3 Visit to WIC breastfeeding support website

Participants were asked whether they had visited the WIC Breastfeeding Support website in the last year. To aid their recall and increase the accuracy of their responses, the question was accompanied by the website URL and a screenshot of the homepage.

#### 2.5.4 Engagement with WIC breastfeeding support website content.

Those who answered “yes” to the question were also asked to indicate which of the four content types they used: (1) read articles, (2) watched videos, (3) clicked links to other webpages from WIC or other government programs, and (4) shared links with friends or family. They were allowed to check all that applied.

#### 2.5.5 Breastfeeding attitude

Four items tapping into general attitudes were culled from the Iowa Infant Feeding Attitude Scale (Mora et al., 1999). The items included “Breastfeeding increases mother‐infant bonding” and “Breast milk is the ideal food for babies.” The responses were captured on a 5‐point Likert scale ranging from 1 (strongly disagree) to 5 (strongly agree), resulting in possible scores between 4 and 20. A higher score reflected a more positive attitude toward breastfeeding. The four‐item subscale was marginally reliable (α_pretest_ = 0.65; α_posttest_ = 0.67).

#### 2.5.6 Breastfeeding self‐efficacy

The Breastfeeding Self‐Efficacy Scale‐Short Form (BSES‐SF) was used to assess breastfeeding confidence (Dennis, 2003) which was defined as “a mother's perceived ability to carry out breastfeeding (Dennis & Faux, 1999).” The 14‐item scale was measured using a 5‐point Likert scale ranging from 1 (not at all confident) to 5 (always confident), resulting in possible scores between 14 and 70. Higher scores indicated higher self‐efficacy. The scale was highly reliable (α_pretest_ = 0.96; α_posttest_ = 0.96).

#### 2.5.7 Breastfeeding social support

A breastfeeding social support scale (Wilson, 2020) was adapted to measure mothers' perceived support. The items addressed informational and emotional domains of social support and the support available to women enrolled in the WIC program. The scale included items such as “I have received support from my family about breastfeeding” and “I have received support from my WIC peer counselor about breastfeeding.” The 7‐item scale was measured using a 5‐point Likert scale ranging from 1 (strongly disagree) to 5 (strongly agree), resulting in possible scores between 7 and 35. Higher scores indicated higher social support. The scale was reliable (α_pretest_ = 0.82; α_posttest_ = 0.81).

#### 2.5.8 Breastfeeding initiation

One question asked whether the respondent was currently breastfeeding their baby. The following question asked whether the respondent ever breastfed the baby. Those who answered no to both questions were coded as “no initiation.” All others were coded as “initiation.”

#### 2.5.9 Breastfeeding duration

For the respondents who answered that they were currently breastfeeding their baby, their baby's age at the time of the survey became breastfeeding duration. The baby's age had been captured in weeks or months by an earlier question. Those who ever breastfed but were not currently breastfeeding their baby were presented with one more question asking how many weeks or months they had breastfed or pumped milk for their baby. Respondents who did not initiate breastfeeding were assigned “0.” Subsequently, the duration was recoded into two categories, “less than three months” and “three months or longer.”

### 2.6 Statistical analysis

H1–H3, RQ1, and RQ2 were analyzed using Chi‐square tests to compare dichotomous variables. H4–H6 were analyzed using one‐way ANCOVA to compare continuous variables while controlling for precampaign measures. All three dependent variables used in the ANCOVA were normally distributed. Intervention message exposure was the independent variable in each of the two‐group comparisons. The ratio of participants in the “no‐low exposure” and “medium‐high exposure” groups was approximately 4:1. The analysis results are summarized in Table 1.

**TABLE 1:** Intervention message exposure effects on campaign‐specific and breastfeeding‐general variables.

		Intervention message exposure		
		No–low	Medium–high	
H1	Campaign tagline	20%	34%	*X* ^2^ = 13.74***
	Campaign logo	18%	31%	*X* ^2^ = 15.16***
H2	WIC website visit	25%	54%	*X* ^2^ = 53.05***
H3	WIC website: read articles	14%	40%	*X* ^2^ = 57.65***
	WIC website: watched videos	16%	32%	*X* ^2^ = 24.33***
	WIC website: clicked links	6%	18%	*X* ^2^ = 29.54***
	WIC website: shared links	1%	9%	*X* ^2^ = 31.22***
H4	Breastfeeding attitude	*M* = 16.51	*M* = 17.26	*F* = 4.5*
H5	Breastfeeding self‐efficacy	*M* = 49.94	*M* = 54.48	*F* = 6.88**
H6	Breastfeeding social support	*M* = 25.11	*M* = 27.37	*F* = 19.21***
RQ1	Breastfeeding initiation	89%	91%	*X* ^2^ = 0.811
RQ2	Breastfeeding duration	73%	76%	*X* ^2^ = 0.677

* for *p* < .05; ** for *p* < .01; *** for *p* < .001.

To answer RQ3 and RQ4 exploring the potential indirect effects of social media intervention on breastfeeding initiation and duration, logistic regression analyses were conducted with posttest responses only. In the regression models, breastfeeding initiation or duration was predicted by intervention message exposure, breastfeeding attitude, self‐efficacy, and social support. Detailed statistics are provided in Table 2. All statistical tests were performed using SPSS.

**TABLE 2:** Regression models predicting breastfeeding initiation and duration.

	RQ3 Breastfeeding initiation	RQ4 Breastfeeding duration
	Exp(B)	Exp(B)
Intervention message exposure	0.885	0.807
Attitude	1.262***	1.160***
Self‐efficacy	1.047***	1.084***
Social support	0.987	0.977
Nagelkerke *R* ^*2*^	0.206	0.346
*N*	757	738
*X* ^*2*^	80.788***	202.982***

*** for *p* < .001; For both regression models, Exp(B)s (odds ratios) are reported.

## 3 RESULTS

### 3.1 H1. Effect on WIC breastfeeding support campaign awareness

A higher percentage of WIC participants with medium–high exposure to the campaign messages (34%) identified the campaign tagline accurately than those with no–low exposure (20%). The difference between the two groups was statistically significant, *X* ^2^ (1, 783) = 13.74, *p* < .001. Similarly, a higher percentage of WIC participants with medium–high exposure to the campaign messages (31%) recognized the campaign logo than those with no–low exposure (18%). The group difference was statistically significant, *X* ^2^ (1, 758) = 15.16, *p* < .001. H1 was fully supported.

### 3.2 H2. Effect on the visit to WIC breastfeeding support website

A higher percentage of WIC participants with medium–high exposure to the campaign messages (54*%*) visited the WIC Breastfeeding Support website than did their counterparts with no–low exposure (25*%*). The difference was statistically significant, *X* ^2^ (1, 806) = 53.05, *p* < .001. H2 was supported.

### 3.3 H3. Effects on engagement with WIC breastfeeding support website content

A higher percentage of WIC participants with medium–high exposure to the campaign messages (40*%*) read articles on the WIC Breastfeeding Support website than their counterparts with no–low exposure (14*%*). The difference was statistically significant, *X* ^2^ (1, 832) = 57.65, *p* < .001. Similarly, a higher percentage of WIC participants with medium–high exposure (32%) watched videos on the WIC Breastfeeding Support website than those with no–low exposure (16%). The difference was statistically significant, *X* ^2^ (1, 832) = 24.33, *p* < .001. Moreover, a higher percentage of WIC participants with medium–high exposure to the campaign messages clicked (18%) and shared links (9%) on the WIC Breastfeeding Support website than the participants with no–low exposure (6% clicked and 1% shared). The difference was statistically significant for those who clicked on links, *X* ^2^ (1, 832) = 29.54, *p* < .001, and shared links, *X* ^2^ (1, 832) = 31.22, *p* < .001. H3 was fully supported.

### 3.4 H4. Effect on breastfeeding attitude

WIC participants with medium–high exposure to the campaign message exhibited more positive attitudes (*M* = 17.26, *SD* = 2.54) than those with no–low exposure (*M* = 16.51, *SD* = 2.68). The difference was statistically significant, *F* (1, 822) = 4.5, *p* = .034. H4 was supported.

### 3.5 H5. Effect on breastfeeding self‐efficacy

Medium‐high exposure to the campaign messages (*M* = 54.48, *SD* = 12.35) was associated with higher self‐efficacy than no‐low exposure (*M* = 49.94, *SD* = 14.65). The difference was statistically significant, *F* (1, 722) = 6.88, *p* = .009. H5 was supported.

### 3.6 H6. Effect on breastfeeding social support

Medium–high exposure to the campaign messages (*M* = 27.37, *SD* = 4.90) was associated with higher perceived social support than no–low exposure (*M* = 25.11, *SD* = 5.52). There was a significant difference in the mean postcampaign support for breastfeeding, *F* (1, 819) = 19.21, *p* < .001. H6 received support.

### 3.7 RQ1. Direct effect on breastfeeding initiation

The percentage of WIC participants who initiated breastfeeding was 91% in the medium–high exposure group and 89% in the no–low exposure group. There was no statistically significant difference in breastfeeding initiation between the two groups, *X* ^2^ (1, 767) = 0.811, *p* = .37. The social media intervention did not increase breastfeeding initiation.

### 3.8 RQ2. Direct effect on breastfeeding duration

The percentage of WIC participants who breastfed for 3 months or longer was 76% in the medium–high exposure group and 73% in the no–low exposure group. The two groups were not statistically different, *X* ^2^ (1, 714) = 0.677, *p* = .41. The social media intervention did not affect breastfeeding duration.

### 3.9 RQ3. Indirect effect on breastfeeding initiation via breastfeeding attitude, self‐efficacy, or social support

Breastfeeding initiation was predicted by social media intervention, breastfeeding attitude, self‐efficacy, and social support. The logistic regression model was statistically significant, *X* ^2^ (4, 757) = 80.79, *p* < .001. As suggested by the RQ1 analysis, the intervention was not a significant predictor of breastfeeding initiation, *OR* = 0.89, 95% CI [0.47, 1.67], *p* = .705. Neither was social support, *OR* = 0.99, 95% CI [0.93, 1.05], *p* = .646. On the other hand, breastfeeding attitude, *OR* = 1.26, 95% CI [1.15, 1.38], *p* < .001, and self‐efficacy, *OR* = 1.05, 95% CI [1.03, 1.07], *p* < .001, were significant predictors. Considered together with the H4 and H5 results, the regression analysis suggested indirect effects of the social media intervention on breastfeeding initiation via improved attitude and self‐efficacy.

### 3.10 RQ4. Indirect effect on breastfeeding duration via breastfeeding attitudes, self‐efficacy, or social support

Breastfeeding duration was predicted by social media intervention, breastfeeding attitude, self‐efficacy, and social support. The logistic regression model was statistically significant, *X* ^2^ (4, 738) = 202.98, *p* < .001. As suggested by the RQ2 analysis, the intervention was not a significant predictor of breastfeeding duration *OR* = 0.81, 95% CI [0.51, 1.28], *p* = .359. Neither was social support, *OR* = 0.98, 95% CI [0.93, 1.02], *p* = .322. On the other hand, breastfeeding attitude, *OR* = 1.16, 95% CI [1.08, 1.25], *p* < .001, and self‐efficacy, *OR* = 1.08, 95% CI [1.07, 1.10], *p* < .001, were significant predictors. Considered together with the H4 and H5 results, the regression analysis suggested indirect effects of social media intervention on breastfeeding duration via improved attitude and self‐efficacy.

## 4 DISCUSSION

A social media intervention was evaluated for its impact on campaign awareness, WIC Breastfeeding Support website use, breastfeeding attitude, self‐efficacy, perceived social support, and breastfeeding behaviors. The intervention met its objectives by generating higher campaign awareness, more visits to the WIC website, more engagement with various elements of the website content, more positive breastfeeding attitudes, higher self‐efficacy, and higher perceived social support. On the other hand, the direct impact on breastfeeding initiation and duration was not statistically significant. Instead, the intervention has shown some promise of effecting behavioral changes by improving attitude and self‐efficacy.

These findings are encouraging for breastfeeding promotion advocates. While a few other programs showed the feasibility and potential benefits of social media‐based interventions in low‐income countries or for underserved women in the U.S., they did not include rigorous testing of the intervention efficacy among their target populations. This social media intervention was the first large‐scale trial since 2011 when the Institute of Medicine Food and Nutrition Board prioritized the adoption of social media in future breastfeeding promotion campaigns. In the absence of established intervention protocols and a robust evaluation framework, the current program advanced the research and practice by filling the gaps and demonstrating the positive outcomes specific to the Campaign as well as broadly related to breastfeeding in general.

At the same time, the lack of direct impacts on breastfeeding initiation and duration reminds us how challenging it is to change breastfeeding behaviors through social marketing campaigns. After completing the 2004–2006 NBAC, the program lead reflected that, in retrospect, increasing breastfeeding was unrealistic for a 2‐year campaign. Given that the current intervention lasted for only half as long, the focus should be on what was improved during the 1 year and how the gains could be leveraged to facilitate the ultimate behavioral changes. The positive relationships between breastfeeding attitude/self‐efficacy and initiation/duration suggest that future interventions should monitor changes in the key psychological constructs and changes in the behavioral outcomes. Future studies could also focus on identifying the threshold level of intervention dosage that prompts significant behaviors changes.

Of the three key psychological constructs, our data showed that self‐efficacy was as strong a predictor of breastfeeding initiation and duration as attitude. The need to emphasize self‐efficacy was another major lesson from the NBAC that the current intervention adopted. On the other hand, the lack of association between perceived social support and breastfeeding initiation/duration illustrates that communication‐based interventions—regardless of the platforms deployed—could become more impactful when supplemented by community‐based support.

Despite these contributions to breastfeeding promotion research and practice, this intervention and its evaluation have limitations. First, the level of intervention message exposure has room for improvement. In this age of audience fragmentation and media saturation, the 4:1 ratio between no–low exposure and medium–high exposure participants was encouraging for a social media intervention. Still, it could become more impactful if the exposure level is increased. Furthermore, the medium–high exposure group was more favorably predisposed to breastfeeding than the no–low exposure group in terms of attitude, self‐efficacy, and social support at the pretest. Selective exposure—people's tendency to seek out, whether consciously or unconsciously, messages that support their pre‐existing views while avoiding the opposite messages—is a well‐documented phenomenon in media research and has been observed in health communication campaign contexts as well (Knobloch‐Westerwick et al., 2013). It would have been ideal to randomly assign WIC mothers to the two exposure levels and compare the two groups in terms of their posttest breastfeeding attitudes and behaviors. In the absence of such an option, we measured the same variables in the pretest and statistically controlled them to identify the differences between the two groups in the posttest that are not attributable to the initial differences. The statistical solution, albeit being a conventional method of addressing the common threat to validity, leaves the findings of this study vulnerable to a possibility that the group differences were due to another variable(s) not controlled in the statistical model. Third, more than two‐thirds of WIC participants who completed the pretest did not complete the posttest, potentially introducing biases to the results. Because there was a 1‐year gap between the pretest and posttest, a lot of women might have left the WIC program after their children became one year old by the end of the intervention and thus could not have been reached by their local offices even though their contact information collected in the pretest was used again to solicit participation in the posttest. With SAs/ITO in charge of the evaluation data collection, it remains unclear how well they were able to perform outreach and recruit participants given the severe staffing shortages caused by the continuing COVID‐19 pandemic. SA/ITOs did not use email to recruit pretest participants, which might also have contributed to the low posttest response rate. It is difficult to ascertain whether and how much of the nonparticipation in the posttest was intentional, as opposed to random, and thus introduced selection bias. However, it is certainly possible that some of the pretest participants grew frustrated with breastfeeding during the intervening period and possibly stopped it altogether, which might have inclined them to ignore the invitation for the posttest survey. If that were to be the case for a significant percentage of the posttest nonresponses, the findings of this study should be understood with the limitation as well. Fourth, there were unusually high numbers of missing answers for breastfeeding initiation and duration. Participants might have felt uncomfortable answering the questions, despite efforts to clarify that participation in the survey would not affect eligibility. The missing answers likely resulted in higher initiation and 3‐month breastfeeding rates than the national statistics. The initiation and 3‐month breastfeeding rates for WIC children reported here were higher than the 84% and 71% rates among children born in 2018 in the U.S. captured in the CDC National Immunization Survey (Center for Disease Control and Prevention, N.D.). Because the focus of this evaluation was on the comparison between the two intervention message exposure groups, the overestimations are not major threats to the validity of our findings. Fifth, the intervention exposure measure was solely based on self‐reporting and could be strengthened by behavioral measures in a more controlled environment. Lastly, the reliability of the attitude measure was marginal. Although we used IIFAS, a validated measure of breastfeeding attitude, the 14‐item measure did not generate a high Cronbach's alpha score and a factor analysis generated four factors with eigenvalues higher than one. With the attitude being a central construct in breastfeeding research, further research is needed.

## 5 CONCLUSIONS

A social media‐based intervention promoting breastfeeding among WIC participants was evaluated for its impact on campaign awareness, use of the WIC Breastfeeding Support website use, breastfeeding attitude, self‐efficacy, perceived social support, and breastfeeding behaviors. The intervention met its objectives by generating higher campaign awareness, more visits to the WIC website, more engagement with various elements of the website content, more positive breastfeeding attitudes, higher self‐efficacy, and higher perceived social support. On the other hand, the direct impacts on breastfeeding initiation and duration were not statistically significant. Instead, the intervention has shown some promise of effecting behavioral changes by improving attitude and self‐efficacy. These findings suggest that thoughtfully designed and implemented social media interventions can effectively deliver messages to their target audiences and positively influence key psychological constructs. Future interventions should consider a longer term program that also integrates community‐based support to accrue the intended behavioral outcomes.

## AUTHOR CONTRIBUTIONS

**Louisiana M Sanchez:** Data curation (equal); formal analysis (equal); writing – original draft (lead). **Sung‐Yeon Park:** Conceptualization (lead); formal analysis (equal); funding acquisition (supporting); investigation (equal); methodology (equal); writing – original draft (supporting); writing – review and editing (lead). **Taya Kohnen:** Data curation (equal); investigation (equal); project administration (equal); validation (equal). **Bret Sarnquist:** Data curation (equal); investigation (equal); project administration (supporting); validation (equal). **Hyo Jin Jeon:** Conceptualization (equal); funding acquisition (supporting); investigation (equal); methodology (equal). **Michelle Granner:** Conceptualization (equal); funding acquisition (supporting); investigation (equal); methodology (equal). **Kelly Morning:** Conceptualization (equal); funding acquisition (supporting); investigation (equal); methodology (equal). **Patricia MacNeil:** Conceptualization (equal); resources (equal); supervision (lead); writing – review and editing (supporting). **Olivia Deavers:** Conceptualization (equal); resources (equal); supervision (supporting); writing – review and editing (supporting). **Valery Soto:** Conceptualization (equal); resources (equal); supervision (supporting); writing – review and editing (supporting). **Elizabeth Christiansen:** Conceptualization (equal); formal analysis (equal); funding acquisition (lead); investigation (equal); methodology (equal); project administration (equal).

## FUNDING INFORMATION

This manuscript reports findings from a cooperative agreement between USDA FNS and the University of Nevada, Reno (UNR) to implement WIC Breastfeeding Support Campaign (Award # WIC‐BFC‐2019‐NV).

## CONFLICT OF INTEREST STATEMENT

There is no conflict of interest to report.

## ETHICS STATEMENT

The Office of Research Integrity at the University of Nevada, Reno waivered an ethics review for the data collection.

## Data Availability

The data that support the findings of this study are available from the corresponding author upon reasonable request.
